# Recruitment and participation of a survey in a public–private primary care setting: experience from the QUALICOPC Malaysia

**DOI:** 10.1017/S1463423620000511

**Published:** 2020-11-20

**Authors:** Masliyana Husin, Norazida Ab Rahman, Xin Ci Wong, Kamaliah Mohamad Noh, Seng Fah Tong, Willemijn Schäfer, Wienke Boerma, Rifat Atun, Sheamini Sivasampu

**Affiliations:** 1 Institute for Clinical Research, National Institute of Health, Ministry of Health, Kuala Lumpur, Malaysia; 2 Department of Public Health, Faculty of Medicine, Cyberjaya University College of Medical Sciences, Selangor, Malaysia; 3 Department of Family Medicine, Faculty of Medicine, University Kebangsaan Malaysia, Selangor, Malaysia; 4 Nivel, The Netherlands Institute for Health Services Research, Utrecht, The Netherlands; 5 Harvard T.H Chan School of Public Health, Harvard University, Boston, Massachusetts, USA

**Keywords:** primary care doctors, primary healthcare, QUALICOPC, recruitment, response rate

## Abstract

**Aim:**

The purpose of this paper is to describe the recruitment strategies, the response rates and the reasons for non-response of Malaysian public and private primary care doctors in an international survey on the quality, cost and equity in primary care.

**Background:**

Low research participation by primary care doctors, especially those working in the private sector, is a challenge to quality benchmarking.

**Methods:**

Primary care doctors were sampled through multi-stage sampling. The first stage-sampling unit was the primary care clinics, which were randomly sampled from five states in Malaysia to reflect their proportions in two strata – sector (public/private) and location (urban/rural). Strategies through endorsement, personalised invitation, face-to-face interview and non-monetary incentives were used to recruit public and private doctors. Data collection was carried out by fieldworkers through structured questionnaires.

**Findings:**

A total of 221 public and 239 private doctors participated in the study. Among the public doctors, 99.5% response rates were obtained. Among the private doctors, a 32.8% response rate was obtained. Totally, 30% of private clinics were uncontactable by telephone, and when these were excluded, the overall response rate is 46.8%. The response rate of the private clinics across the states ranges from 31.5% to 34.0%. A total of 167 answered the non-respondent questionnaire. Among the non-respondents, 77.4 % were male and 22.6% female (*P* = 0.011). There were 33.6% of doctors older than 65 years (*P* = 0.003) and 15.9% were from the state of Sarawak (*P* = 0.016) when compared to non-respondents. Reason for non-participation included being too busy (51.8%), not interested (32.9%), not having enough patients (9.1%) and did not find it beneficial (7.9%). Our study demonstrated the feasibility of obtaining favourable response rate in a survey involving doctors from public and private primary care settings

## Background

A strong primary care system contributes to universal health coverage by providing affordable and equitable access to quality health services (Atun, 2004). To strengthen primary care, research on primary care is essential as it provides evidence for benchmarking and quality improvement initiatives (Schäfer *et al.*, 2019). However, prior works have shown that primary care doctors are hard to engage in research (VanGeest *et al.*, 2016), and primary care doctors’ response rate has remained static despite increasing evidence on strategies to increase recruitment (Creavin *et al.*, 2011). Common reasons for non-participation from primary care doctors were time constraint, existing burden on administrative work (Kaner *et al.*, 1998), concerns with patient confidentiality, scepticism in applicability of research results (Rosemann and Szecsenyi, 2004) and lack of interest and irrelevant research topics (Tong *et al.*, 2018). Moreover, studies that require greater demands on primary care doctors’ time and resources would likely face greater challenges in eliciting response. There is also an issue of ‘gatekeepers’ whereby administrative staff filters telephone calls and letters that reaches the clinics to avoid time intrusion to the primary care doctors (Scott *et al.*, 2011).

In an attempt to evaluate the performance of the primary care, the international QUALICOPC (Quality and Cost of Primary Care) study was initiated and conducted in several countries. It consists of surveys among primary care doctors and patients to gather data for comparison and quality improvement of primary care service delivery (Schäfer *et al.*, 2011). Among the 34 countries that have participated in QUALICOPC, the response rates vary between 2% and 85%. The variation in response rate could be attributed to survey load as well as the differences in incentives around the funding mechanism of primary care in each country (Groenewegen *et al.*, 2016). Notably, among countries where primary health care is primarily funded through public sources (e.g., social health insurance, governmental budgets), the average response rate of QUALICOPC was 34%, whereas among countries where primary health care is funded either through exclusively private sources (e.g., out of pocket or private health insurance) or mixed public–private sources, the response rate was 10% on average (Groenewegen *et al.*, 2016).

The Malaysian arm of the QUALICOPC study was conducted in 2016 for a sound and comprehensive studies on primary care services and performance reporting. Malaysia is the first country in Asia to adopt and to adapt the QUALICOPC study. Primary care in Malaysia comprises a dual sector system with the public clinics being exclusively funded from governmental budgets and private clinics being exclusively funded from patient’s out of pocket payments or third-party payers (Harvard TH Chan School of Public Health, 2016). Against this background, the Malaysian QUALICOPC study surveyed both public and private clinics. This paper aims to describe the recruitment strategies, the response rates and the reasons for non-response of QUALICOPC study in Malaysia. In addition to highlighting the recruitment strategies for both the public and private primary care doctors, we would also like to describe challenges we faced in engaging primary care doctors to participate in the research and factors that influence survey recruitment, which we hope can be a lesson for countries with similar dual-sector primary care system.

## Methods

### Setting

Primary care in Malaysia is provided by both the public and private clinics. In 2014, there were 911 (12%) public health clinics and 5646 (88%) private health clinics (Sivasampu *et al.*, 2015). Public primary clinics were originally intended to provide maternal and child health services in the rural areas. However, since the 1980s, it has evolved into the establishments of public health clinics with extended services in curative, preventive, promotive and rehabilitative care in both urban and rural areas (Jaafar *et al.*, 2013). On the other hand, private primary care clinics exist mostly in the urban areas as either solo or group practices. While solo practices are owned by individual doctors, group practices could either be a co-owned by several doctors or owned by a corporation that employs the doctors and takes care of administrative burdens. While the number of private primary care clinics outnumber that of public clinics, approximately 60% of the outpatient visits are to public (Institute for Public Health (IPH), 2015). The majority of doctors in both public and private clinics are registered medical officers with basic medical training. Only a minority holds postgraduate training in family medicine and other medical disciplines (Sivasampu *et al.*, 2015).

### QUALICOPC study

The QUALICOPC study is a multi-country study that evaluates quality, cost and equity in primary care (Schäfer *et al.*, 2011). Malaysia is the 35^th^ country to participate in this study. Institute for Clinical Research (ICR), a research institute under the National Institutes of Health, Ministry of Health Malaysia, was appointed as the national coordinator for the Malaysian QUALICOPC study under the umbrella of the Malaysian Health System Research (MHSR) study.

QUALICOPC used a cross-sectional study design to collect data on primary care practices, primary care doctors and their patients with four questionnaires – the General Practitioner (GP) questionnaire, the Patient Experience questionnaire, the Patient Values questionnaire and the Practice questionnaire (Schäfer *et al.*, 2013). The GP questionnaire examines the workload and services delivered; the patient experience questionnaire examines patient’s experience during a visit to the primary care clinics; the patient value questionnaire measures patient’s preference and the practice questionnaire records response rate and practice characteristics.

All questionnaires, adapted from the QUALICOPC Europe, were designed to measure the structure, process and outcome of primary care in 10 dimensions, namely the governance, economic condition, workforce development, access, continuity of care, coordination, comprehensiveness, quality, efficiency and equity. The questionnaires were made available in three languages: English, Malay and Mandarin. The QUALICOPC questionnaires in English were translated into Malay language and Mandarin (back-translated to ensure accuracy) with minor adaptation to the Malaysian setting. Details on the QUALICOPC study questionnaires are described in detail elsewhere (Sivasampu *et al.*, 2016). Questionnaires were completed either through interview or self-administration. All self-administered questionnaires were checked, and any missing information was obtained with active follow-up on the same day.

The study was undertaken in public and private primary care clinics from five main states – Kuala Lumpur, Selangor, Kelantan, Sabah and Sarawak. These five states were selected out of 14 states in Malaysia to provide a good representation of the population in different regions of the country. The general sample size as per the international QUALICOPC study protocol was 220 doctors from 220 primary care clinics in each country (Schäfer *et al.*, 2011), with doctor being the unit of sampling and the unit of analysis. In our study, doctor remains as the unit of analysis but due to the lack of regular updates on practice location in the doctor’s register, sampling was performed at the clinic level. The clinic-level sampling ensured reliability and efficiency as the clinic register is regularly updated for regulatory purposes. In order to compare between public and private primary care, the target sample size for Malaysia was set to 220 public and 220 private primary care doctors. However, prior works suggested that the average response rate would be lower among private primary care doctors at approximately 30% (Teng, 2014); therefore, we oversampled the private clinics by additional 70% to obtain a sample size for private clinics of 730.

The list of primary care clinics was obtained from the Ministry of Health Malaysia through the Family Health Development Division for public clinics and the Private Medical Practice Control Division for private clinics. Clinics were selected through multi-stage stratified random sampling from two separate sampling frames of public and private clinics. Stratification was carried out on state and urban/rural classification, and clinics were randomly sampled proportionate to the number of eligible clinics across stratum. If a sampled clinic had more than one doctor working on that day of data collection, only one primary care doctor per clinic would be selected to participate in the study; in the public primary care clinics, the doctor was selected by simple random sampling, whereas in the private primary care clinics, the doctor was selected by convenient sampling. For each of the participating doctor, 10 patients seen by them on the day of data collection were invited to respond to the patient experience questionnaire.

### Recruitment of primary care doctors

The recruitment and data collection were conducted in two phases. Phase 1 was carried out in public sector from August to October 2015. Phase 2 was carried out in private sector from February to June 2016. Data collection processes involved visits to clinics by fieldworkers for interviews. In both phases, the same data collection method was employed, but recruitment methods differ slightly between public and private primary care doctors. Recruitment strategies were based on literature and past experiences, as summarised in Table 1.

**Table 1.** Recruitment strategies

Sector	Sampling of clinics and doctors	Period of data collection	Endorsement from relevant associations or authorities	Survey invitation	Non-monetary incentives
Public	Stratified random sampling from five state, one doctor selected from each clinic by random sampling	- August to October 2015	- Ministry of Health	- Invitation letter	- CPD points
				- Introductory	- Certificate of participation
Private	Stratified random stratified sampling from five states; one doctor selected from each clinic by convenience sampling	- February to June 2016	- Ministry of Health	- Invitation letter	- CPD points
					- Certificate of participation
			- Malaysia Medical Association	- Telephone contact by fellow doctors	- Gift cards
			- Large chain network of primary care clinics	- Face-to-face briefing	- CME training
				- News article and postcard in bulletin of the medical professional society^a^ workshops	

^a^Malaysian Medical Association monthly bulletin.

CME = continuous medical education; CPD = continuing professional development.

#### Public primary care doctors

Given that the public primary care clinics are accountable to the respective State Health Departments of Ministry of Health, we sought their endorsement on the survey, which subsequently provided support by inviting doctors in public clinics to participate in the study. This endorsement was included in the invitation letter provided to all public primary care doctors. Endorsements have been shown to be effective in increasing participation in survey among primary care doctors (Kottke *et al.*, 1990; Asch *et al.*, 2000; Flanigan *et al.*, 2008; Pit *et al.*, 2014; Parkinson *et al.*, 2015). Additionally, introductory workshops where the team briefed the doctors about the study procedures and the dates for data collection were carried out. This strategy involved personal pre-contact to enhance recruitment (Pit *et al.*, 2014). In terms of data collection, while there have been advocates for mailed surveys, we opted for a face-to-face interview to reduce the potential burden to the doctors, especially in obtaining responses from patients in the patient experience questionnaire (Hoddinott and Bass, 1986). Patient recruitment was essential in ensuring completeness of the data collection in the facilities and to consider it as a one-unit response. Here, QUALICOPC aims for 1800 patient experience and 220 patient value to represent a reliable country estimate. Non-monetary incentive in the form of continuous professional development points and certificate of participation were also offered (Pit *et al.*, 2014). Participation was entirely a decision by the doctors. They were informed about their rights to refuse and were assured of no implication if they decided not to participate.

#### Private primary care doctors

We approached the Malaysian Medical Association (MMA), which is the main representative body for registered medical doctors in Malaysia, for endorsement towards the QUALICOPC study. One of the benefits of approaching doctors through the professional association is that it takes advantage of the formal and informal relationships between the association and the doctors in their network (Asch *et al.*, 2000). The MMA helped in promoting the study by making announcement on the Malaysian QUALICOPC through news articles in the MMA monthly bulletin with attached postcards that contained the link to the study website, and continuing medical education (CME) sessions organised specifically for QUALICOPC (Pit *et al.*, 2014). This created awareness among the private doctors about the legitimacy of this research and acts as a form of ‘social recruitment’ as explained by Kottke *et al.* (1990). With the endorsements, we contacted the doctors by telephone to confirm eligibility and interest in participation. This allows personal pre-contact as mentioned earlier. Additionally, for clinics that are part of a larger clinic chain (e.g., QUALITAS, Mediviron), we contacted the central management office of the corporation to obtain endorsement. This limits the potential gatekeeping barriers faced when approaching their chains (Scott *et al.*, 2011). Once eligibility and interest in participation were confirmed, a follow-up email containing study brochures and invitation letters (Flanigan *et al.*, 2008; Pit *et al.*, 2014) was sent to the clinics. Telephone calls that went unanswered after more than two attempts were abandoned. Recruitment was halted after achieving the targeted sample size of 220. Similarly, face-to-face interview was the method of data collection. Visit to the practices where the doctors have agreed to participate were made based on their preferred date on a working day with the normal patient panel. On top of that, non-monetary incentive in the form of continuous professional development points, certificate of participation and gift token were also offered.

### Data collection

The QUALICOPC research team composed of 10 sub-teams of one to two fieldworkers each, led by a team leader. Face-to-face interview with the primary care doctor took place at the practice followed by interviews of 10 patients seen by the participating doctor. Patient interview involved the administration of one patient value questionnaire alongside nine patient experience questionnaires on the same day. In certain district, particularly in Sabah and Sarawak, where local dialect is more commonly spoken in communities, fieldworkers who spoke the local dialect were assigned for the interviews. Doctors and patients who refused to participate were asked to complete the non-response questionnaire that recorded their basic demographic information and reason for non-participation. In private clinics with small number of patients per day, patient interviews were conducted for more than one day to obtain sufficient number of responses from patients.

Questionnaires were administered through tablet computers using an offline survey application. The user interface for these electronic questionnaires has been designed to aid in minimising response error by respondents. The captured information was then uploaded to a central database via a secure internet connection.

### Data analysis

Response rate was calculated as the number of completed GP questionnaire divided by total doctors sampled. Chi-square test was used to demonstrate statistical difference in characteristics between the respondents’ and the non-respondents’. All analysis was conducted using R version 3.4.0 in R studio (R Core Team, Vienna, Austria).

## Results

A total of 221 public primary care doctors and 239 private primary care doctors participated in the QUALICOPC study. Table 2 summarises the characteristics of the primary care doctors and practice characteristics. All public primary care doctors completed the questionnaire through interview with fieldworkers, whereas private doctors completed the questionnaire through interview (79.2%) and self-administration (20.8%). The median duration to complete the questionnaire was 44 minutes (interquartile range: 35.3–58.8 minutes) for public doctors and 58.5 minutes (interquartile range: 32.0–102.0 minutes) for private doctors (data not shown).

**Table 2.** Socio-demographic characteristics of primary care doctors and practice characteristics in public and private clinics

	Public (*n* = 221)	Private (*n* = 239)
**DOCTORS CHARACTERISTICS**		
Sex, *n* (%)		
** **Male	85 (38.5)	157 (65.7)
** **Female	136 (61.5)	82 (34.3)
Age, *n* (%)		
** **25–34 years	192 (86.9)	21 (8.8)
** **35–44 years	24 (10.9)	50 (20.9)
** **45–54 years	5 (2.3)	76 (31.8)
** **55–64 years	-	55 (23.0)
** **≥65 years	-	37 (15.5)
Job title, *n* (%)		
** **Medical officer^a^	163 (73.7)	191 (79.9)
** **Family medicine specialist^b^	3 (1.3)	12 (5.0)
** **Medical officer in charge^c^	55 (24.8)	-
** **Other specialist^d^	-	36 (15.0)
Born in Malaysia, *n* (%)		
** **Yes	213 (96.4)	226 (94.6)
Self-employed or salaried, *n* (%)		
** **Salaried	221 (100.0)	80 (33.5)
Hours worked per week, median (IQR)	40 (40–40)	40 (35–40)
PRACTICE CHARACTERISTICS		
Group practice, *n* (%)	185 (83.7)	131 (54.8)
Location, *n* (%)		
** **Urban	101 (45.7)	224 (93.7)
** **Rural	120 (54.3)	15 (6.3)
Patient population above average, *n* (%)		
** **Elderly	51 (23.1)	45 (18.8)
** **Socially disadvantaged	155 (70.1)	72 (30.1)
** **Ethnic minority	47 (21.3)	22 (9.2)
Extended hours, *n* (%)		
** **Open after 6.00pm	162 (73.3)	189 (79.1)
** **Open during weekends	161 (72.9)	223 (93.3)
Facilities in practice, *n* (%)		
** **Laboratory facilities	156 (70.6)	11 (4.6)
** **X-ray facilities	56 (25.3)	67 (28.0)

^a^Medical Officer: registered doctors with basic medical training; ^b^family medicine specialist: doctor with postgraduate training in family medicine; ^c^Medical Officer in charge: senior medical doctor in charge of entire clinic (public clinics only); ^d^other specialists: doctor with other postgraduate qualifications, for example Diploma in Family Medicine, occupational health and dermatology.

Abbreviations: IQR = interquartile range; *n* = count; % = percentage.

**Figure 1.**
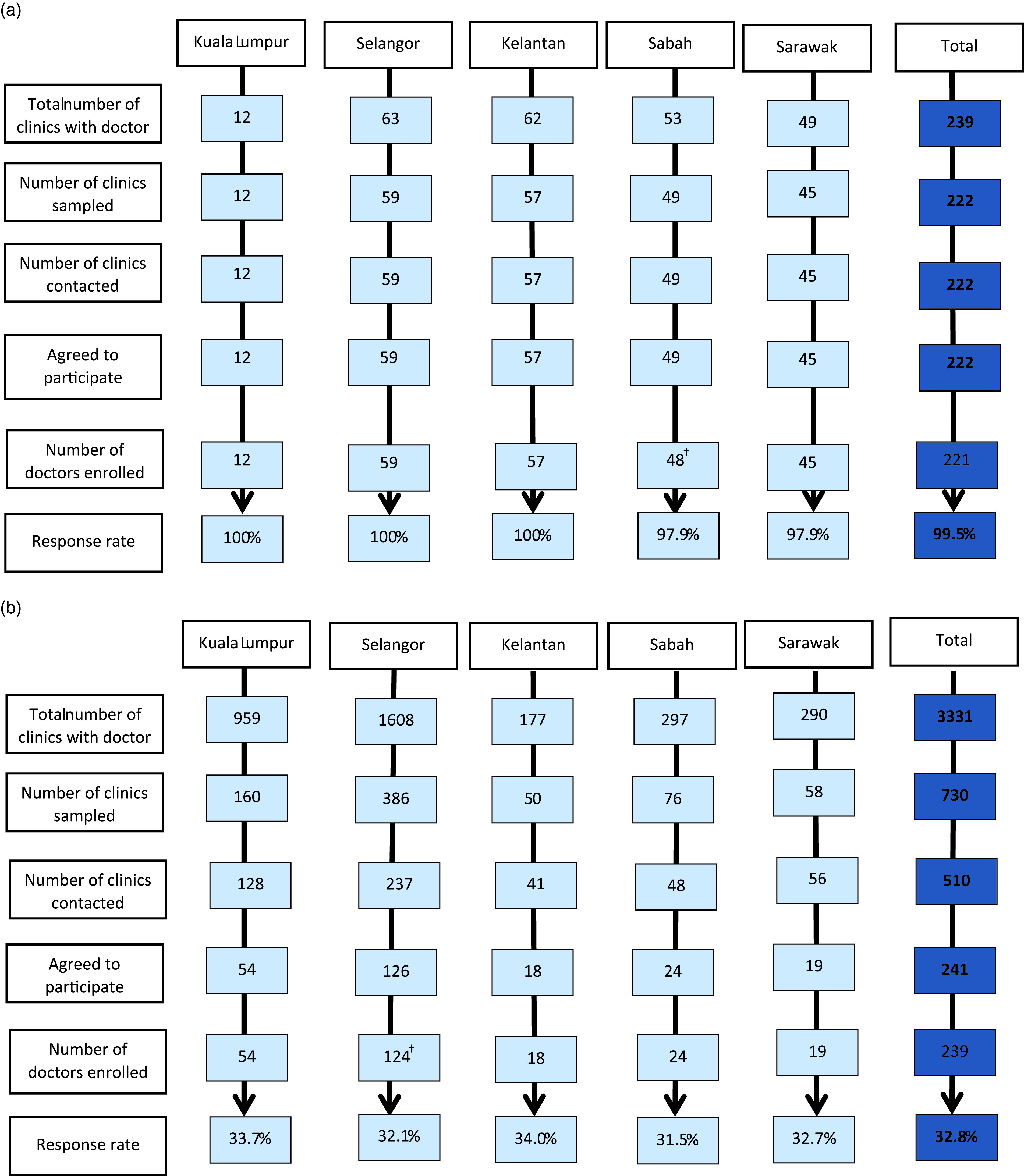
Flow diagram of QUALICOPC recruitment of primary care doctors. 1a- Flow diagram of QUALICOPC recruitment of primary care doctors from public primary care clinics. Response rate was calculated by the number of doctors enrolled divided by total number of clinics sampled. The primary care doctors must complete the GP questionnaire to be considered enrolled. †One primary care doctor from Sabah who had initially agreed was unable to complete the GP questionnaire on the day of data collection. 1b- Flow diagram of QUALICOPC recruitment of primary care doctors from private primary care clinics. †Two primary care doctors from clinics in Selangor had initially agreed but were unable to complete the GP questionnaire on the day of data collection. GP = general practitioner.

Figure 1 shows flow diagram for recruitment of public (Figure 1a) and private clinics (Figure 1b) for overall and by states. For the public clinics, all 222 primary care doctors sampled for the survey agreed to participate. However, only 221 out of 222 public primary care doctors completed the GP questionnaire bringing the overall response rate to 99.5 %.

For the private clinics, 510 clinics were successfully contacted by telephone out of the 730 clinics in the sample. The remaining 220 clinics were unreachable by phone. Of these, 241 private primary care doctors agreed to participate with 239 doctors completed the GP questionnaire yielding a response rate of 32.8%. Nevertheless, after exclusion of the uncontactable clinics, the overall response rate is 46.8%. The response rate of the private primary care doctors across the states ranges from 31.5% to 34.0%. All states achieved the targeted sample size.

### Non-response

In total, we had 269 private clinics that refused to participate. However, only 167 private doctors returned partially completed non-response questionnaire. The most common cited reasons by the doctors to decline participation were being too busy (51.8%). Other reasons included not interested in the study (32.9%), did not feel like they have enough patients to be interviewed (9.1%) or did not find it beneficial (7.9%). Among the non-respondents, 77.4 % were male and 94.5% were from urban areas. The practice location of urban–rural did not differ between respondents and non-respondents who refused participation. However, there was a higher proportion of male and older doctors. There were also more doctors in Sarawak that answered the non-respondents questionnaire among non-respondents (Table 3).

**Table 3.** Respondents and non-respondents characteristics for private clinics

Characteristics	Respondents (*n* = 239)	Non-respondents (*n* = 167)	*P*-value
Sex, *n* (%)			0.011
Male	157 (65.7)	127 (77.4)	
Female	82 (34.3)	37 (22.6)	
Age, *n* (%)			0.003
25–34 years	15 (6.3)	-	
35–44 years	50 (20.9)	16 (11.4)	
45–54 years	75 (31.4)	35 (25.0)	
55–64 years	57 (23.8)	42 (30.0)	
≥65 years	42 (17.6)	47 (33.6)	
State, *n* (%)			0.016
Selangor	124 (51.9)	61 (37.2)	
WPKL	54 (22.6)	49 (29.9)	
Sarawak	19 (7.9)	26 (15.9)	
Sabah	24 (10.0)	15 (9.1)	
Kelantan	18 (7.5)	13 (7.9)	
Strata, *n* (%)			0.743
Urban	224 (93.7)	155 (94.5)	
Rural	15 (6.3)	9 (5.5)	

## Discussion

In this paper, we presented the recruitment strategies, the response rates and the reasons for non-response for Malaysian QUALICOPC, a locally-adapted international study on quality and costs of primary care. With the three pronged strategies of endorsement, survey invitation and incentives, we were able to obtain favourable response rates from both public and private clinics. The response rate from the public clinics was close to 100%, surpassing that obtained in earlier national primary care survey (Sivasampu *et al.*, 2015) and the highest among all countries that have participated in QUALICOPC to date. Meanwhile, the response rate from the private clinics (32.8%) which are not reimbursed by any public funds, despite being lower than that of the public clinics, was higher than those obtained in QUALICOPC studies in Australia, Canada and several European countries. The achievement of the target sample sizes of 220 public clinics and 220 private clinics was the first among all countries that participated in the QUALICOPC study. We attribute the achievement to our recruitment strategies, which we believe would provide useful lessons for other countries planning to embark on similar studies to benchmark primary care in the future. Our study is one of the few QUALICOPC studies that captured the characteristics of non-respondents. Below, we reflect on the recruitment processes and data collection critically in terms of challenges, strategies and reasons for non-participation to generate new insight for future studies.

### Recruitment strategies and challenges

The appointment of ICR as a central coordinator for all states and sectors facilitated the whole conduct of the study due to our previous experience in performing national survey in primary care. The role of national coordinator for a large survey has shown to be an effective strategy in increasing response rate (Groenewegen *et al.*, 2016). Our sampling method had an overall objective of obtaining the targeted sample size as stipulated by QUALICOPC. Due to the absence of a reliable national doctor register, recruitment entailed obtaining the lists for both public and private clinics from the authorities. The existing list for the private sector was matched to our own list of private clinics obtained from earlier surveys. This was followed by the intensive process of identifying whether clinics were still operational. In addition, a strategy of oversampling was used in order to achieve the target numbers of private clinics required.

In the public sector, endorsement by the Ministry of Health expedited the recruitment process. This allowed diffusion to the Family Health Development Division and State Health Departments, which the public primary care doctors are managed by. The response rates may have been lower otherwise as public primary care doctors would likely decline participation in research studies considering the high patient workload in most public clinics. Although recruitment of doctors in the private sector also had the endorsement by the Ministry of Health, we postulate that the approach through professional association (MMA) played a larger part. This is due to the fact that private primary care doctors practice independently without any links to the government. By liaising through the professional association, articles about the study and postcards were easily promoted through their monthly bulletin. Free teaching in the form of CME sessions were made available to private primary care doctors in Kuala Lumpur and Selangor. The CME session was based on their preferred topics that they found useful for their routine practice. This strategy was deployed in Kuala Lumpur and Selangor as they have the most number of primary care doctors sampled and our past experience showed that it was harder to recruit primary care doctors in these regions. Endorsement through network managers for private primary care doctors that works under group practices was also used. Agreement from their top management was obtained before the doctors were approached by telephone contacts. We found that this strategy lowered primary care doctors’ resistance towards the survey.

The second strategy was to invite doctors through personal contact instead of invitations through email or postal letter (Asch *et al.*, 2000; Flanigan *et al.*, 2008). It was possible to organise introductory workshops for the public primary care doctors as the initial endorsement by the ministry of health functioned as a mandate. This allowed the study coordinators to gather doctors in the same states together to introduce and brief about the study. On the other hand, it would be challenging to gather the private primary care doctors together in state level introductory workshops due to the opportunity costs to the private primary care doctors and the practice operating times varies between clinics. Hence, private primary care doctors had to be invited by personal contact through telephone (Heywood *et al.*, 1995). Although labour and cost intensive, benefits of these methods outweigh its drawbacks as our previous experiences showed that we were able to get better response rates. It is believed that this was a more effective method to use due to the design of QUALICOPC which is a multi-actor survey whereby primary care doctors consent to include patients into the survey had to be obtained (Schäfer *et al.*, 2011). This method allowed a clear exchange of information on the study and helps to establish trust with doctors by explaining the purpose of the research, why their participation is important and the plan for data collection. Moreover, contact with familiar and trusted research groups can be invaluable in the recruitment process. For instance, primary care doctors who previously participated in past surveys by ICR are already familiar with the ICR research group and reported positive experience and good rapport; this facilitated the recruitment of clinics and primary care doctors for the QUALICOPC study. Hence, our response rate is higher compared to other QUALICOPC study that used mail-outs or email request (Wong *et al.*, 2015).

QUALICOPC required a two-step participation status, and during the recruitment phase, these details were exchanged. Once a doctor agreed to participate, a condition that 10 of his/her patients to also participate in the survey to complete the questionnaire were highlighted and informed to all concerned. Yet, past research has shown that this increases the chances of non-response due to concerns about patient confidentiality (Hummers-Pradier *et al.*, 2008). Another strength to highlight was that our trained fieldworkers managed to convince most primary care doctors and patients to participate in the study and collection of data by interview approach also eased the process. Despite this, we still observed refusal from 269 primary care doctors. We managed to capture the reason for non-participation from 62% out of all non-respondents whom cited lack of time and lack of interest. We believe that the possibility of losing income when participating in a survey could also be a factor to non-participation. This may explain the higher response rate for the salaried public primary care doctors compared to the private primary care doctors.

### Data collection processes, challenges and applied strategies

Data quality was maintained through face-to-face interview of primary care doctors by fieldworkers to ensure completeness of the survey during data collection. This was a trade-off to the resources spent on a face-to-face interview. The visits to the clinics not only facilitated the doctor’s interview but it also permitted patient recruitment and interview to be conducted on the same day. The inability to recruit the minimum number of patients caused the practice to be considered a ‘unit’ non-response. The entire data collection process was carried out by the fieldworkers, thereby minimising the burden of the clinic workers for any task related to the study. To reduce interviewer bias, the task of interviewing the primary care doctors was given to trained team leaders. Furthermore, we also allow flexibility to cater to doctors preferences on when they want to be interviewed, besides option for self-administration of the questionnaire. It was observed that most of them preferred assisted interviewer-based administration.

In addition to the standard QUALICOPC questionnaires, we added a set of non-response questionnaire to capture details of non-responders. Demographic and practice pattern characteristics were assessed by comparing between respondents and non-respondents. Despite concerted efforts by researchers, only minimal response and information were obtained from non-responders. From the available data, our result concurs with the literature, whereby there are more male non-respondents in the older age group (Stocks and Gunnell, 2000). There were more doctors in Sarawak that answered the non-respondents, questionnaire and a possible explanation is that there were a higher proportion of clinics from other states that were not captured through the non-response questionnaire.

### Strengths and limitations

The strength of this study is that the methodology and strategies being used in recruiting primary care doctors were supported with a report on the response rates and data on non-respondents to measure potential bias. The limitations are the rate of non-response that could arise from the multi-actor design and minimal data on non-response. Bias can also arise from sampling of clinics to reach doctors due to the absence of a national doctor’s register. In the case of QUALICOPC, the main focus is the interplay between patient experiences and characteristics of the primary care doctor’s practice, so these biases are considered less relevant.

## Conclusion

Our experience in conducting the QUALICOPC study demonstrated the feasibility in obtaining favourable response rate in a national survey involving doctors from both public and private primary care setting by adapting strategies from the literature to the local context. Specifically, the favourable response rates in our study could be attributed to having a national coordinator and as well as our three-pronged strategies in obtaining endorsement from professional associations, state health departments and private clinic managers, personalised invitation for recruitment, face-to-face interview and non-monetary incentives. In light of our experience, we recommend future research involving primary care doctors to adapt these strategies to obtain better response rates. We also recommend the inclusion of non-response analysis to make valid inferences about the target population.
